# Complete Acid Ceramidase ablation prevents cancer-initiating cell formation in melanoma cells

**DOI:** 10.1038/s41598-017-07606-w

**Published:** 2017-08-07

**Authors:** Michele Lai, Natalia Realini, Marco La Ferla, Ilaria Passalacqua, Giulia Matteoli, Anand Ganesan, Mauro Pistello, Chiara Maria Mazzanti, Daniele Piomelli

**Affiliations:** 1 Drug Discovery and Development, Istituto Italiano di Tecnologia, Genova, 16163 Italy; 2 Pisa Science Foundation, Pisa, 56121 Italy; 3 Retrovirus Center and Virology Section, Department of Translational Research, University of Pisa, Pisa, Italy; 4 Departments of Anatomy and Neurobiology, Pharmacology and Biological Chemistry, University of California, Irvine, CA, 92617 USA; 5 Department of Dermatology, University of California, Irvine, California, 92868 USA

## Abstract

Acid ceramidase (AC) is a lysosomal cysteine hydrolase that catalyzes the conversion of ceramide into fatty acid and sphingosine. This reaction lowers intracellular ceramide levels and concomitantly generates sphingosine used for sphingosine-1-phosphate (S1P) production. Since increases in ceramide and consequent decreases of S1P reduce proliferation of various cancers, AC might offer a new target for anti-tumor therapy. Here we used CrispR-Cas9-mediated gene editing to delete the gene encoding for AC, *ASAH1*, in human A375 melanoma cells. *ASAH1*-null clones show significantly greater accumulation of long-chain saturated ceramides that are substrate for AC. As seen with administration of exogenous ceramide, AC ablation blocks cell cycle progression and accelerates senescence. Importantly, *ASAH1*-null cells also lose the ability to form cancer-initiating cells and to undergo self-renewal, which is suggestive of a key role for AC in maintaining malignancy and self-renewal of invasive melanoma cells. The results suggest that AC inhibitors might find therapeutic use as adjuvant therapy for advanced melanoma.

## Introduction

Acid ceramidase (*N*-acylsphingosine deacylase, #EC 3.5.1.23; AC) is a lysosomal cysteine hydrolase encoded by the *ASAH1* gene, which catalyzes the conversion of ceramide into fatty acid and sphingosine^1^. This reaction lowers intracellular ceramide levels and concomitantly generates the substrate needed for sphingosine-1-phosphate (S1P) production by sphingosine kinases^1^. Ceramide and S1P serve important, but often opposing, functions in normal and cancer cell signaling: most notably, ceramide decreases whereas S1P increases the proliferation of a variety of cancer cells in cultures^2–4^. Thus, maintaining a dynamic equilibrium between the intracellular levels of ceramide and those of S1P, a primary function of intracellular AC activity, has emerged as a potential objective for cancer therapy^5, 6^. Three complementary lines of evidence support this idea. First, *ASAH1* transcription is abnormally elevated in various cancers *–* including melanoma, glioma and prostate cancer^3, 5, 7–9^. This upward regulation is thought to confer resistance to apoptosis and stimulate proliferation and invasiveness of cancer cells^7, 10, 11^. Indeed, it has been suggested that the cytotoxic actions of certain drugs (e.g. dacarbazine, anthracyclines) depend on the ability of these agents to increase the intracellular levels of ceramide^12–14^. Second, pharmacological inhibition of AC activity sensitizes prostate cancer cells to the effects of radiation^15^ and fenretinide^16^, promotes Fas-induced apoptosis in head-and-neck cancer^17^, increases daunorubicin cytotoxicity in hepatoma cells^18^ and enhances cytotoxicity of several classes of chemotherapeutic drugs in colon cancer^19^ and melanoma cells^5, 8^. Finally, siRNA-guided silencing of the *ASAH1* gene reduces hepatocellular carcinoma growth *in vivo* ^18^ and synergizes with silencing of Akt to enhance death in a variety of cancer cell lines *in vitro* ^20, 21^.

In addition to these roles in apoptosis and chemoresistance, ceramide is also implicated in the regulation of cellular senescence. The levels of this lipid molecule are significantly elevated in senescent cells and application of its cell-permeant analog C6 accelerates senescence in colon adenocarcinoma and lung cancer cells^13, 22^. Ceramide is also involved in the control of stem-cell differentiation and embryo development^23^. Mutant mice in which *ASAH1* is deleted by homologous recombination do not survive beyond the embryonic 2-cell stage and undergo apoptotic cell death, while treatment with exogenous S1P rescues embryonic AC-null stem cells and permits their survival^24^.

The role of AC in balancing ceramide and sphingosine/S1P levels is reasonably well established. The consequences of the long-term suppression of this balance by removal of AC are unknown, because all experiments conducted thus far have relied upon gene silencing or pharmacological approaches that do not achieve complete and prolonged AC suppression^19, 20, 25^. To overcome this limitation, in the present study we used CrispR/Cas9-mediated gene editing to remove the *ASAH1* gene and its protein product from A375 melanoma cells, which are known for their high invasiveness and self-renewal capabilities^26^.

## Materials and Methods

### Cell cultures

Human epithelial melanoma A375 cells were purchased from American Type Culture Collection (Manassas, VA) and cultured in Dulbecco’s Modified Eagle’s Medium (DMEM) supplemented with 10% fetal bovine serum (FBS), 2 mM L-glutamine and antibiotics (penicillin, streptomycin) at 37 °C and 5% CO_2_.

### Crisp/Cas9 design, transfection, and transduction–A

CrispR/Cas9 gRNA targeting *ASAH1* exon 6 –ATAAATACATTCGTGCCAAGTGG - was designed and cloned into pKLV-U6gRNA(BbsI)-PGKpuro2ABFP (#50946Addgene, MA, USA) following a standard protocol^27^. This protocol provides experimentally derived guidelines to select the target sites and evaluate cleavage efficiency and off-target activity. Transduction was performed using HIV-1 packaging and Vescicular Stomatitis virus pseudotyped envelope. This vector contains Blue Fluorescent Protein (BFP) and, as outlined above, gRNA targeting exon 6. We have used a multiplicity of infection (MOI) of 1, as described^28^. The A375 cell line was first transduced with the lentiviral vector. Three days after transduction, A375 cells were sequentially diluted in 96-well plates to isolate clones expressing BFP and gRNA. BFP-positive clones were further transfected with a U6Ex6pspCAS9-GFP plasmid (#48138 Addgene, MA, USA) bearing a gRNA targeting exon 2 – GGACTAAGGCGACGCAACTC - using JetPEI reagent (Polypus transfectionTM, Illkrich, France) and following manufacturer’s instructions. After 48 h, the cells were sorted by flow cytometry. Deletions and cleavage activity were monitored by nested PCR, 5 days after sorting, using two primer pairs as follows: forward out ACTTTGAAATCCAACCCG, forward in GGAGGAAACACAGCCGCTT, reverse in CCACCACCTGCATAATTTTT, reverse out. CGAAGAGGTTGCTGAATT. Off-target activity was measured in 293 T using Surveyor Nuclease Assay (IDT, Coralville, Iowa, USA) following the manufacturer’s protocol. The phenotype recovery of *ASAH1-*null cells was assessed using a commercially available plasmid containing the *ASAH1* cDNA under control of the CMV promoter (#RG212434 Origene, Rockville, MD). Transfection efficiency was approximately 50%, as assessed by FACS analysis.

### RNA isolation, cDNA synthesis and real-time quantitative PCR

Total RNA was extracted 17 days after sorting, using the RNeasy Mini Kit (Qiagen, Venlo, Netherlands) following manufacturer’s protocol. Samples were treated with DNase supplied in the kit and cDNA synthesis was performed using 100 ng of purified RNA and the Pico PCR cDNA Synthesis Kit (Clonetech, Mountain View, CA), according to the provided protocol. First-strand cDNA was amplified using the iQ SYBR Green SuperMix (Life Technologies, Carlsbad, CA). *ASAH1* primer sequence: forward AGTTGCGTCGCCTTAGTCCT; reverse TGCACCTCTGTACGTTGGTC. Quantitative PCR was performed in a 96-well PCR plate and run at 95 °C for 10 min followed by 40 cycles, each cycle consisting of 15 sec at 95 °C and 1 min at 60 °C, using a CFX96 Thermal Cycler (Touch™ Real-Time detection System, BioRad). Primers used to monitor expression of senescence-related genes were obtained from BioRad, those to detect apoptosis were from Qiagen (RT² Profiler PCR Array System - PAHS-012A). Data analysis was performed to determine relative gene expression and stability compared to two different housekeeping genes (glyceraldehyde 3-phosphate dehydrogenase, GAPDH and hypoxanthine-guanine phosphoribosyltransferase, HPRT) and using the on-line software developed by BioRad and Qiagen. Briefly, relative expression of genes of interest was calculated by the equation 2^-ΔCt^, where ΔCt was calculated by subtracting the Ct value of the geometric mean of the housekeeping genes from the Ct value for the genes of interest. Real Time reactions to evaluate the stem-cell profile were performed using the following primers:

**GAPDH** F- CGC TCT CTG CTC CTC CTG TT R -CCA TGG TGT CTG AGC GAT GT; **ABCB1** F- TAT CAG CAG CCC ACA TCA TCA R- CCA AAT GTG ACA TTT CCT TCC; **ABCB5** F- GCT GAG GGA TCC ACC CAA TCT R- CAC AAA AGG CCA TTC AGG CT; **ABCG2** F- GAG CCT ACA ACT GGC TTA GAC TCA A R- TGA TTG TTC GTC CCT GCT TAG AC; **ALDH1A1** F- GCA TCC AGG ATT TTT GTG GA R- TCC CAC TCT CAA TGA GGT CAA; **ALDH1A3** F- GCA TGA GCC CAT TGG TGT CT R- CGC AGG CTT CAG GAC CAT**; CD133** F- CAG AGT ACA ACG CCA AAC CA R- AAA TCA CGA TGA GGG TCA GC; **CD166** F- TGA TCT CCG CCA CCG TCT TCA G R- CTC TTT TCA TCA CTG ATC CTT GCA; **CXCR6** F-CCA GAT GCC CTT CAA CCT CA R- CAG GCT GAC AAA GGC; **NANOG** F- ACC TTG GCT GCC GTC TCT GG R- AGC AAA GCC TCC CAA TCC CAA ACA; **SOX2** F- CCC CCC TGT GGT TAC CTC TTC R- TTC TCC CCC CTC GAG TTG G; **SOX10** F- CTT CAT GGT GTG GGC TCA G R- TGT AGT CCG GGT GGT CTT TC.

### Immunocytochemistry, senescence and apoptosis assays

Cells (10^4^/well) were seeded, 10 days after sorting, on glass chamber slides. Fixed in 4% paraformaldehyde for 10 min and permeabilized in 0.1% Triton × 100-PBS for 15 min. After blocking with 5% goat serum in 0.1% Triton × 100-PBS for 1 h, cells were incubated with anti-AC primary antibody (1:200, Sigma-Aldrich, Saint Louis, Missouri) overnight at 4 °C. Bound primary antibodies were detected with the avidin-biotin complex detection system (AbCam, Cambridge, UK). Nuclei were stained with haematoxylin (Diapath, Martinengo, Italy).

To assess senescence, 17 days after sorting, cells were washed in phosphate-buffered saline (PBS), fixed with 2% paraformaldehyde at room temperature for 5 min and again washed with PBS. Cells were then incubated with fresh senescence-associated stain solution [1 mg/mL 5-bromo-4-chloro-3-indolyl P3-D-galactoside (X-Gal), 40 mM citric acid/sodium phosphate pH 6.0, 5 mM potassium ferrocyanide, 150 mM NaCl, 2 mM MgCl2] at 37 °C and in the absence of CO_2_. Staining was detectable from 2–4 h and maximal in 12–16 h. To detect apoptotic bodies, the terminal deoxynucleotidyl transferase-mediated dUTP nick-end labeling (TUNEL) method was utilized with an ApoMark DNA Fragmentation Detection Kit (Funakoshi, Tokyo, Japan). Images were collected with a Zeiss LSM 800 confocal microscope with a 60X magnification and 1.4 numerical aperture objective lens.

### Flow cytometry: senescence, apoptosis, and cell cycle assay

A typical marker of apoptosis-mediated cellular self-destruction is the activation of nucleases that eventually degrade the nuclear DNA into fragments of approximately 200 base pairs in length^29^. The presence of such laddered DNA was investigated by labeling the DNA strand breaks using APO-BrdU™ TUNEL Assay Kit (Invitrogen, Waltham, MA). Senescence was studied by flow cytometry, performed 20 days after CRISPR/Cas9 ablation, using a fluoreporter lacZ/Galactosidase quantitation Kit (Life Technologies), which stains the cells with a β–galactosidase substrate that is hydrolyzed to a blue fluorescent compound in senescent cells. A375 cells treated with scrambled gRNA and empty plasmid backbone were used as controls. Cell cycle assays were performed using a Cyflow CUBE 8 Sorter (Partec, Kobe, Japan). Briefly, 17 days after the CRISPR/Cas9 ablation, A375 cells were detached from 6-well plates by tripsin treatment, pelleted by centrifugation at 300x*g* for 5 min and washed in PBS. The cells were then fixed in cold 70% ethanol at 4 °C for 30 min, washed twice with PBS, pelleted again and treated with RNase (100 μg/mL) and propidium iodide (50 μg/mL). Data were analyzed using the FSC Express 4 software (DeNovo™ Software, Glendale, CA).

### Cell invasion assay and soft agar assay

Invasion assays were performed using Bio Coat Matrigel invasion chambers (BD Biosciences, Franklin Lakes, USA). Cells were trypsinized, resuspended in serum-free medium DMEM and counted by Trypan-blue exclusion method. An equal number of live cells were then plated in the bottom chamber containing 10% FBS as chemoattractant and incubated at 37 °C for 48 h. Invading cells were fixed in methanol and stained with 0.2% crystal violet. After drying overnight, cells were counted using the ImagePro 6.2 software (Media Cybernetics, Warrendale, PA). Soft agar assays were performed as described^30^, in parallel with the invasion assay. Briefly, 3 × 10^4^/well cells were seeded in triplicate into 6-well plates, stained with 0.2% crystal violet and counted one week later using the ImageJ software (http://imagej.nih.gov/ij/). These experiments were assessed 30 days after CRISPR/Cas9 ablation.

### Proliferation assay

A375 cells (10^4^/well), 17 days after CRISPR/Cas9 ablation, were seeded in 96-well plates with 0,1 mL complete DMEM and allowed to adhere overnight. WST-1 [3-(4,5-dimethyl-2-thiazolyl)-2,5-diphenyl-2H-tetrazolium bromide, 10 µl] buffer from Quick Cell Proliferation Assay Kit (Biovision, San Francisco, California) was added to each well and incubated at 37 °C for 1 h. Absorbance (420 nm) was measured with a μQUANT multiplate reader (Bio-Tek Instruments, Bejing, China).

### Tumor-sphere formation assay

Tumor spheres are solid and spherical formations derived from proliferation of one cancer initiating cell. Tumor-spheres are easily distinguishable from single or aggregated cells. To determine the ability to form tumor-spheres, cells (200/well) were kept for one week in DMEM (10%FBS, high glucose 4.5 g/l,Thermo Fisher, MA, UK) and then cultured in the presence of serum-free DMEM F12, under non-adherent conditions by precoating the 96-well plates with 2% 2-hydroxyethyl methacrylate (poly-HEMA, Sigma). This experiment was started after 30 weeks the CRISPR/Cas9 ablation.

### Lipid extraction

Lipids were extracted according to Bligh and Dyer^31^. Samples were transferred to glass vials and liquid-liquid extraction was performed using a chloroform:methanol mixture (1:2 v/v, 2 mL) with final 0.1% trifluoroacetic acid (TFA), and spiked with internal standards (i.s.). After mixing for 30 s, chloroform (0.6 mL) and water (0.6 mL) were sequentially added and the samples were vortexed after each addition. The samples were centrifuged after 20 days the RT-PCR screening, for 15 min at 3,500x*g* at 4 °C. After centrifugation, the aqueous (upper) and organic (lower) phases were separated by a protein disk. The organic phase was transferred to glass vials. The aqueous fraction was extracted again with chloroform (1 mL). Both organic phases were pooled, dried under N_2_ and residues were dissolved in methanol/chloroform (9:1 v/v; 0.1 mL) and transferred to glass vials for analyses. *LC-MS analyses* - Samples were analyzed by LC-MS using an Acquity UPLC system coupled with Xevo triple-quadrupole mass spectrometer (Waters) as previously described^32^.

### Statistical analysis

GraphPad Prism software V5.03 (GraphPad Software, Inc., USA) was used for statistical analysis. Data were analyzed using the Student’s *t*-test or 2-way ANOVA for multiple comparisons. Differences between groups were considered statistically significant at values of p < 0.05. Results are expressed as mean ± S.E.M of at least 3 independent experiments.

## Results

### CrispR/Cas9 deletion of the *ASAH1* gene

**Figure 1:**
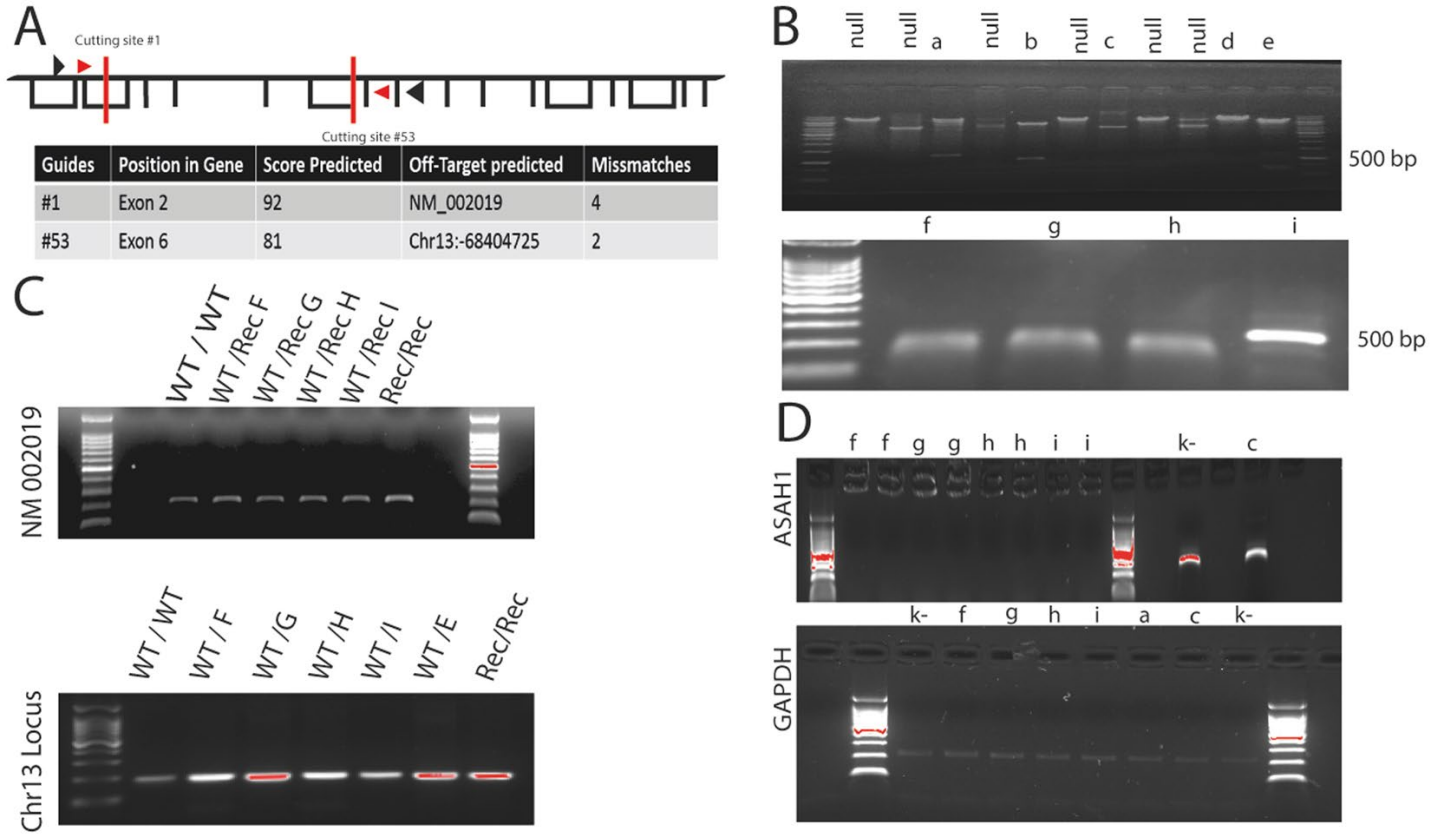
Deletion of the *ASAH1* gene in A375 melanoma cells. (**A**) Schematic structure of the *ASAH1* gene. Red bars indicate gRNA recognition sites, arrows indicate the annealing of nested PCR primers. Predicted guide score and possible off-target sites are also shown. (**B**) Nested PCR analysis of the *ASAH1* gene showing events ofdeletion in heterozygosis (*a, b, c, e*), in homozygosis (*f, g, h, i*). (**C**) Surveyor Nuclease Assay on two loci indicated in panel (**A**) demonstrating the lack of detectable off-target activities of the guides utilized in the present study. (**D**) RT-PCR analysis shows lack of *ASAH1* transcription in deleted clones. *ASAH1* transcripts are still present in clones with single insertion/deletion (InDel) mutations (c).

We used the CrispR/Cas9 system to delete the *ASAH1* gene in A375 melanoma cells. In its standard application, this technique disrupts coding sequences by targeting one critical site in a gene of interest^27^. Here, to ensure total removal of *ASAH1*, we targeted two sites in parallel and selected clones for double cuts and consequent deletion events. We designed gRNAs to guide Cas9 towards two critical exons of the *ASAH1* gene. Figure 1A illustrates the structure of this gene and a schematic summary of guides predicted using an online tool (http://crispr.mit.edu/). Among hundreds available, two guides were chosen based on their anticipated high selectivity and low off-target activity. Figure 1B shows the nested PCR products obtained from various clones of A375 cells: 16 such clones displayed deletion in homozygosis and were used for further experiments (see, for example, clones *f, g, h, i*, Fig. 1B *)*. Clones that showed deletion in heterozygosis were discarded (e.g., clones *c* and *d*, Fig. 1B). A surveyor nuclease assay was performed on two loci (Fig. 1A) to identify off-target activity: no cuts were detected, indicating that neither site was affected by cleavage (Fig. 1C). RT-PCR analyses of *ASAH1*-null clones, collected five days after gene editing, confirmed that *ASAH1* was no longer transcribed (Fig. 1D).

### CrispR/Cas9 ablation of the AC protein

**Figure 2:**
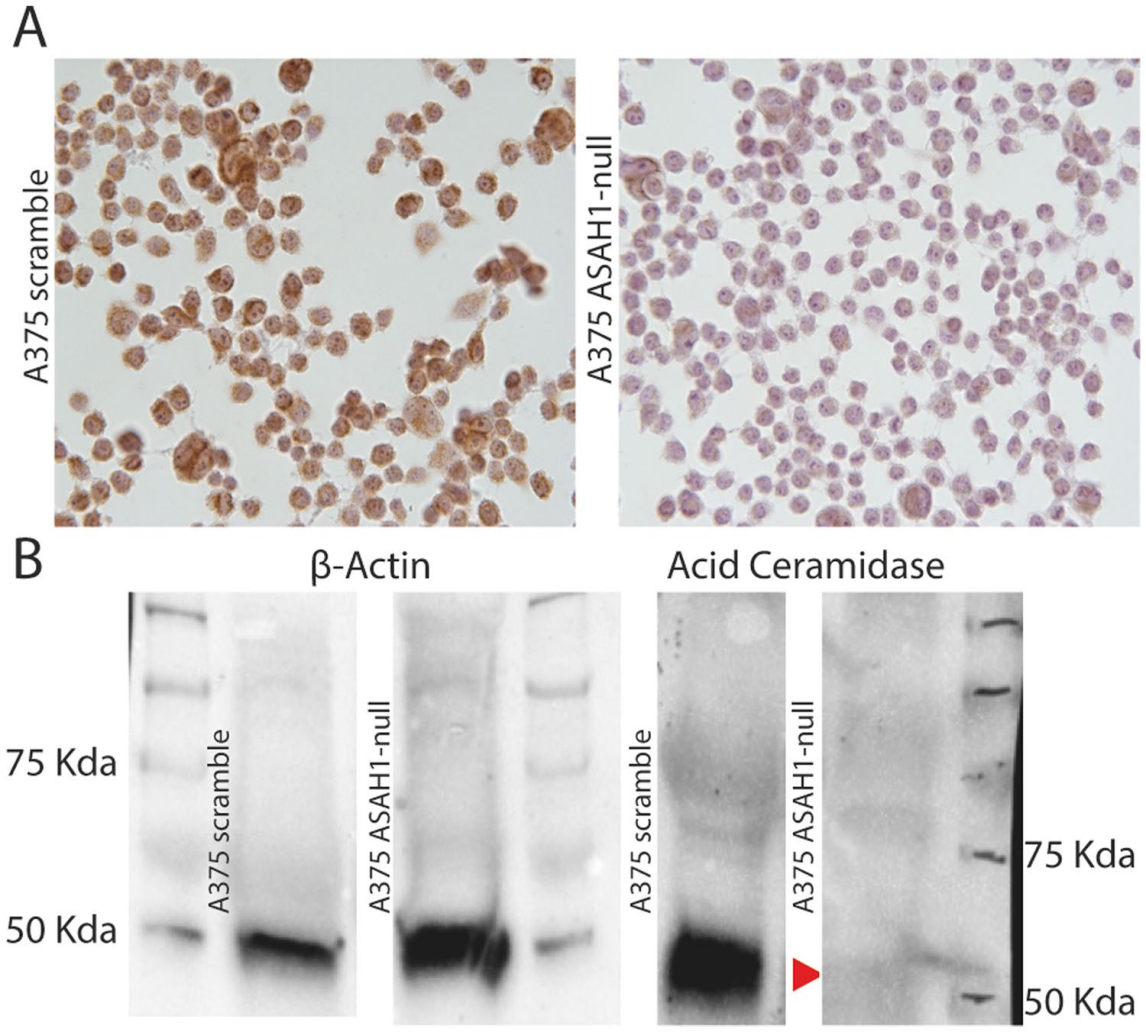
AC deletion in A375 melanoma cells. (**A**) Immunocytochemical analysis showing AC expression in control scramble-treated (left) and *ASAH1*-null (right) A375 cells. (**B**) Western blot analyses demonstrate the presence of AC in control scramble-treated but not in *ASAH1*-null cells. Arrow indicates AC. ß-actin was used as loading control.

Next, we assessed the effect of CrispR/Cas9 gene editing on AC protein levels. Five days after editing, A375 cells were either fixed with paraformaldehyde or lysed, and the presence of AC was assessed by immunocytochemistry or Western blot, respectively. The results show that AC expression was intact in naïve and scramble-treated cells, but was suppressed in *ASAH1*-null cells (Fig. 2A,B).

### AC ablation blocks G_1_/S cell cycle progression

**Figure 3:**
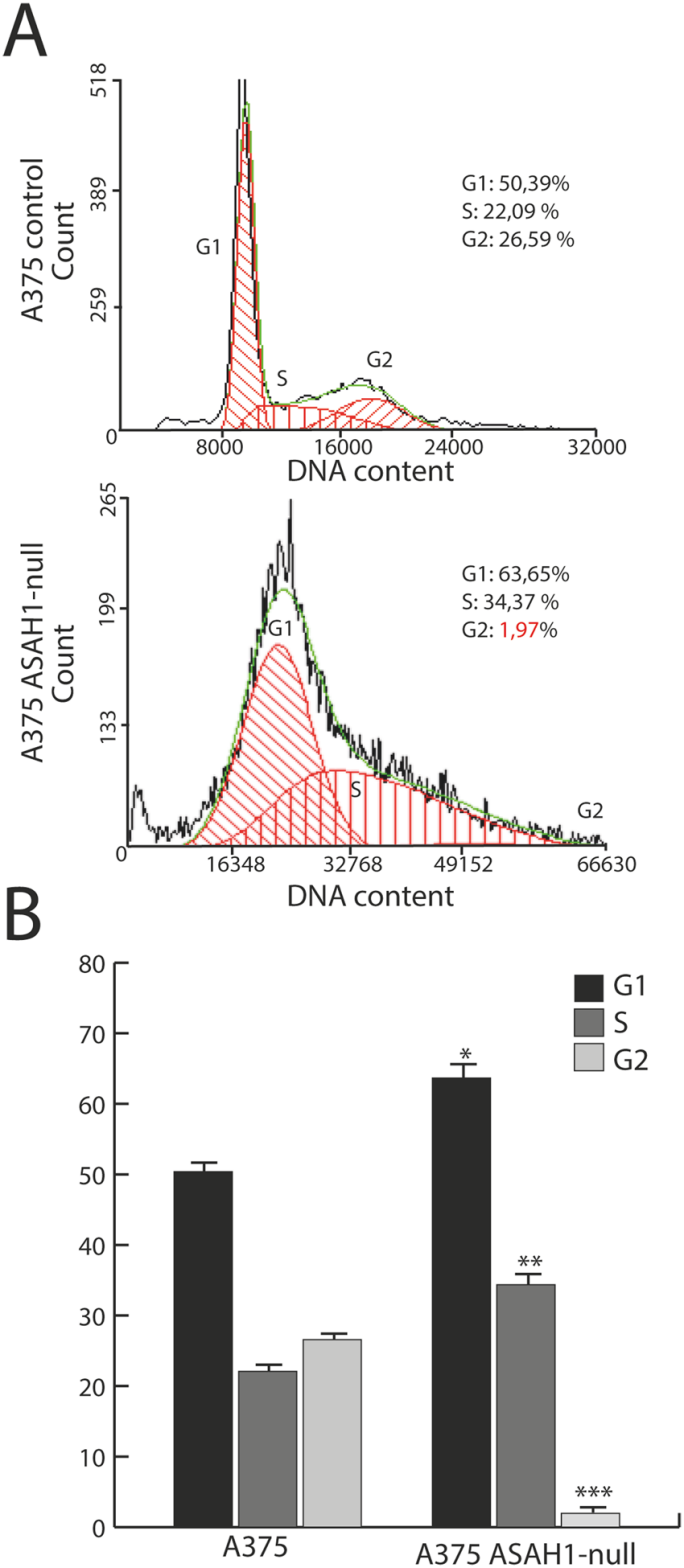
AC deletion stops G1-S cell cycle progression. (**A**) Cell cycle progression was assessed using propidium iodide and flow cytometry analysis. Dot plots show different distribution between G1, S and G2 phases in control (scramble-treated, top) versus *ASAH1*-null cells (bottom). (**B**) Statistical analysis of the cell cycle distribution was performed using the Student’s *t* test; *p < 0.05, ** p < 0,01,***p < 0.001,. Experiments were performed 17 days after CRISPR/Cas9 ablation.

The cell-permeant ceramide analog C6 causes cell cycle arrest in G_1_/S by up-regulating in a dose-dependent manner the kinase inhibitor p27and by modulating retinoblastoma (Rb) protein function during G_2_/M progression^33^. We assessed the impact of AC ablation on the cell cycle using flow cytometry. As shown in Fig. 3A,B, only 2% of *ASAH1*-null cells entered the G2 phase, compared to 25% scramble-treated control cells. The remaining *ASAH1*-null cells were found in G1 (63%) or S (35%) phase. The finding that 98% of *ASAH1*-null cells are slower in completing the cell cycle confirms the key role of AC in regulating cell cycle progression.

### AC ablation promotes senescence

**Figure 4:**
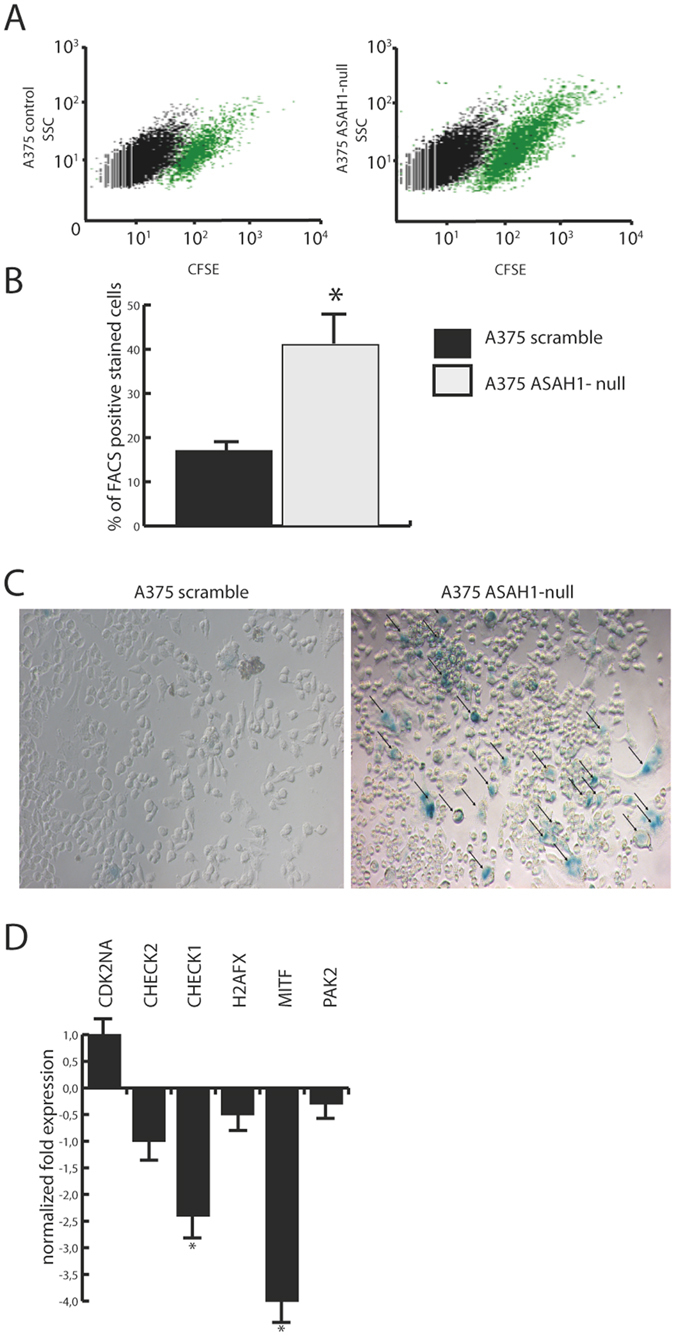
AC deletion accelerates senescence. (**A**) Flow cytometry of senescence-associated β-galactosidase on control (scramble-treated, left) and *ASAH1*-null A375 cells. (**B**) Number of β-gal positive cells in the experiments illustrated in (**A**). Results are expressed as mean ± SEM, n = 3, **p < 0,01, Student’s *t* test. (**C**) β-galactosidase (β-gal) staining of control (scramble-treated, left) and*ASAH1*-null A375 cells (right). (**D**) Real-Time PCR of key genes involved in senescence and cell cycle progression in control (scramble-treated) or *ASAH1*-null cells. Results are expressed as mean ± SEM, n = 3, with each experiment performed in technical and biological triplicates: experiments were performed using three replicates each and repeated three times, *p < 0.05, **p < 0,01,***p < 0.001, Student’s *t* test. Experiments were performed 20 days after CRISPR/Cas9 ablation.

Exogenous C6 accelerates senescence in human fibroblasts^34^. Flow cytometry experiments in *ASAH1*-null cells confirmed this finding and showed that approximately 38% of the cells were positive to β-galactosidase versus 18% scramble-treated control cells (Fig. 4A,B). Similarly, *ASAH1* deletion was accompanied by the appearance of a phenotype characterized by senescence-like cell morphology and accumulation of senescence-associated β-galactosidase (Fig. 4C). Furthermore RT-PCR quantification of 20 mRNAs encoding for genes involved in the induction of senescence (S. Table 1) revealed profound changes (Fig. 4D) in the expression of micropthalmia-associated transcription factor (*MITF*), which controls the DNA damage response and a lineage-specific senescence program in melanoma^35^, and *CHK1*, which impacts various stages of the cell cycle, including the S phase, the G2/M transition and the M phase^36^.

### AC ablation causes apoptosis

**Figure 5:**
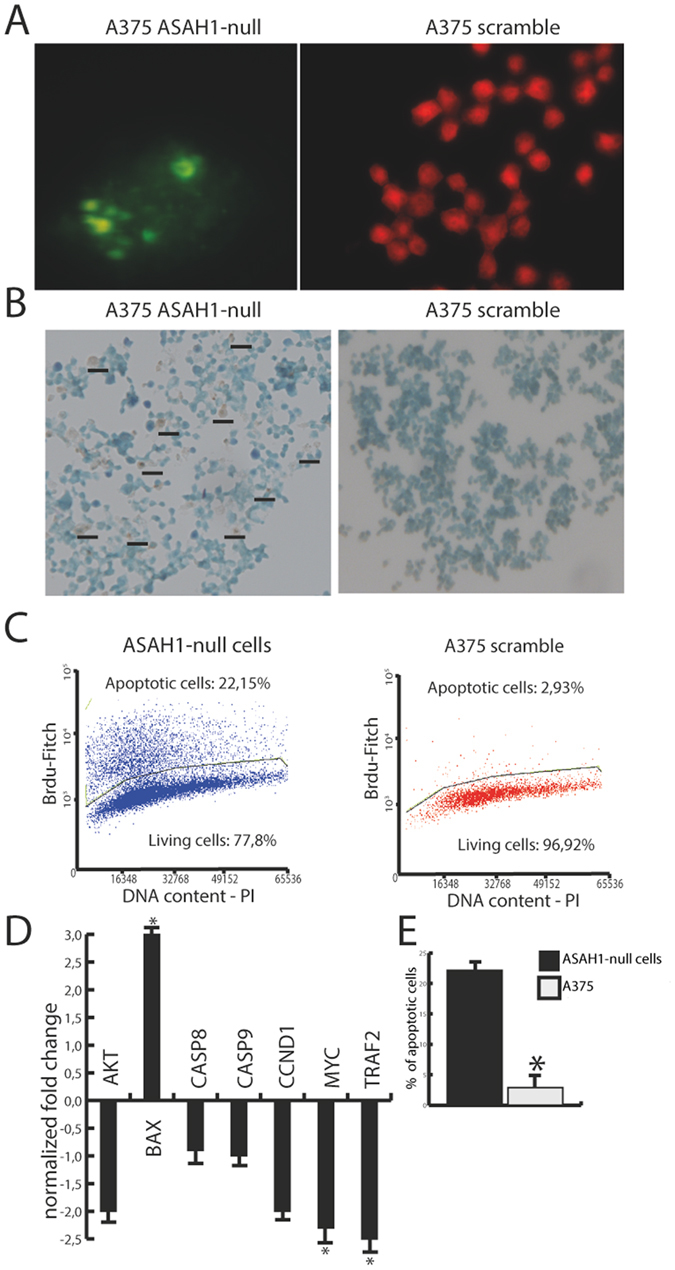
AC deletion causes apoptosis. (**A**) ApoBRDU nuclear staining in *ASAH1*-null (left) and control (scramble-treated) cells (right). (**B**) Immunostaining show the presence of apoptotic bodies in A375 *ASAH1*-null but not in control (scramble-treated). (**C**) Flow cytometry analysis after ApoBRDU staining shows increased apoptotic events in *ASAH1*-null cells after 10 days in culture. (**D**) Real-Time PCR quantification of gene transcripts related to apoptosis in *ASAH1*-null and control cells. Results are expressed as mean ± SEM, n = 3, with each experiment performed in technical and biological triplicates: experiments were performed using three replicates each and repeated three times. *p < 0.05, **p < 0,01,***p < 0.001, Student’s *t* test. (**E**) Statistical analysis of positive marked nuclei after ApoBRDU staining, results are expressed as mean ± SEM, n = 3, with each experiment performed in technical and biological triplicates: experiments were performed using three replicates each and repeated three times. *p < 0.05, **p < 0,01,***p < 0.001, Student’s *t* test. Experiments were performed 17 days after CRISPR/Cas9 ablation.

In alternative to senescence, treatment with exogenous ceramide can also promote apoptosis^33, 37^. This finding was confirmed in *ASAH1*-null cultures by fluorescence microscopy, which showed early blebbing of nuclei (Fig. 5A), as well as by immunocytochemistry studies, where extensive presence of apoptotic bodies was detected in these cultures (Fig. 5B). Flow cytometry experiments revealed higher apoptosis in *ASAH1*-null cells, 22% of which stained for ApoBRdU compared to 3% scramble-treated control cells (Fig. 5C–E). The impact of AC deletion on apoptosis was confirmed by RT-PCR analysis of a panel of 89 genes involved in this process as well as in cell cycle control (S. Table 1). As shown in Fig. 5D, *ASAH1* deletion significantly down-regulated key genes implicated in cell cycle progression and cell survival. Among the surveyed genes, a significant reduction in expression was observed with *TRAF2* (p < 0.012), *MYC* (p < 0.048) and *CYCLIN D1* (p < 0.044), with a trend of reduction for *AKT1* (not statistically significant). In contrast, the ceramide activated proapoptotic factor *BAX* ^38^ was upregulated by *ASAH1* deletion (p < 0.028). No significant changes were seen in the remaining gene transcripts profiled in this experiment (S. Table 1).

### AC ablation prevents tumor cancer-initiating cell formation and self-renewal

**Figure 6:**
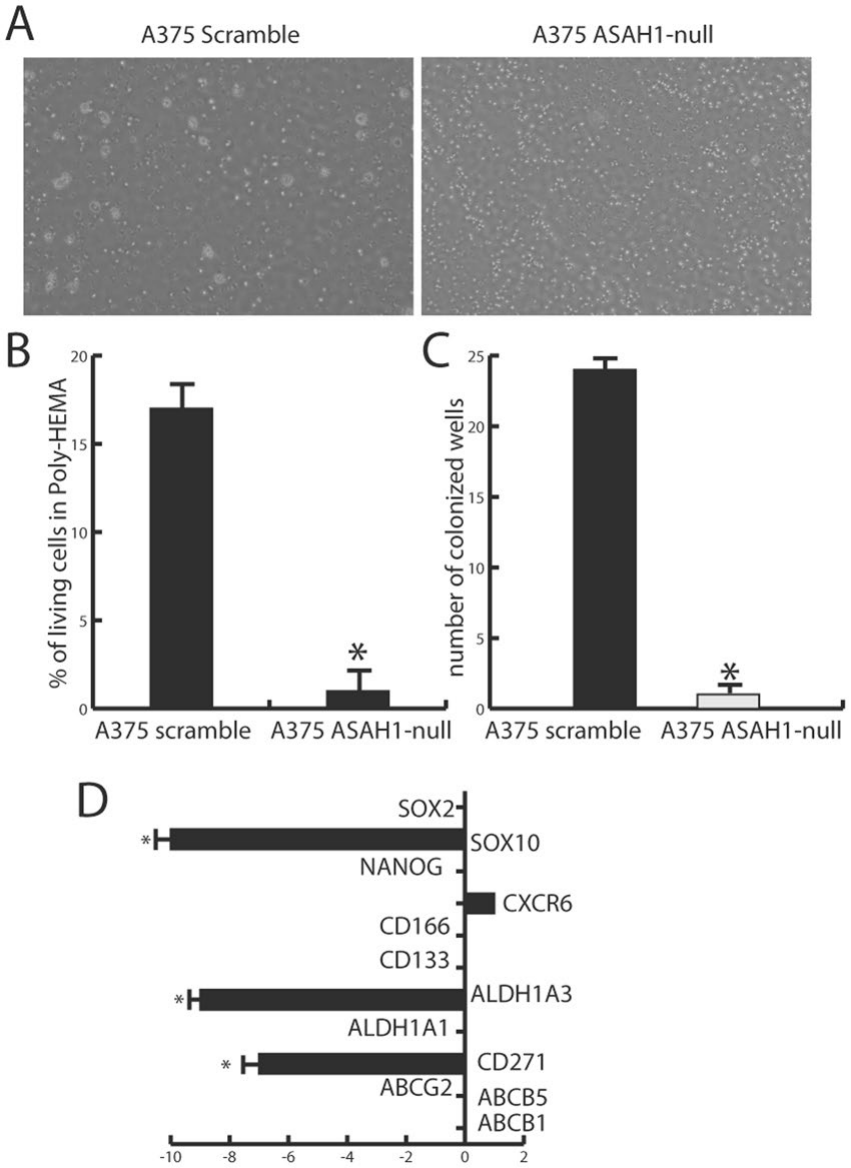
AC deletion suppresses cancer-initiating cell formation and self-renewal. (**A**) Cancer-initiating cell formation on Poly-HEMA-coated plates and vitality assays in control (scramble-treated, left) and *ASAH1*-null A375 cells (right). (**B**) Viability of cells in Poly-HEMA plates. *p < 0.05, ** p < 0,01,***p < 0.001; Student’s *t* test. (**C**) Self-renewal assay in control (scramble-treated) and *ASAH1*-null A375 cells: no wells were colonized by *ASAH1*-null cells indicating suppression of self-renewal capabilities. *p < 0.05, ** p < 0,01,***p < 0.001, Student’s *t* test. (**C**) Real-time PCR showing downregulation of *SOX10*, which is consistent with the observed downregulation of *MITF*. Two crucial genes related to cancer initiating cells formation and metabolism of xenobiotics were downregulated, *CD271* and also *ALDH1A3*. Results are expressed as mean ± SEM, n = 3, with each experiment performed in technical and biological triplicates: experiments were performed using three replicates each and repeated three times. *p < 0.05, **p < 0,01,***p < 0.001, Student’s *t* test. Poly-HEMA and self-renewal experiments were performed after 30 days the CRISPR/Cas9 ablation. RNA extraction for Real-Time PCR was performed 35 days after CRISPR/Cas9 ablation.

Cancer-initiating cells contribute in important ways to the heterogeneity and tumorigenesis of melanoma^39^, but the possible role of AC in the proliferation and differentiation of this subpopulation of cells is still unclear^24^. To address this question, we tested the ability of *ASAH1*-null cells to aggregate into tumor spheroids when cultured in poly-HEMA-coated plates^40^. The results show that scramble-treated control cells form a substantial number of spheroids, whereas *ASAH1*-null cells completely lack this capacity (Fig. 6A,B). To test the ability of *ASAH1*-null cells to undergo self-renewal, we repeated the experiment seeding individual cells in complete DMEM after poly-HEMA selection. Control A375 cells colonized an average of 28 wells, whereas virtually no wells were colonized by the *ASAH1*-null clones (Fig. 6C). Finally, we used RT-PCR to evaluate possible changes in melanoma markers induced by *ASAH1* deletion. No alterations were seen in the levels of *SOX2*, *CD133*, *CD166*, *ALDH1A1* and *ABC*. By contrast, profound downregulations were observed with *CD271* (7 folds), *SOX10* (5 folds) and *ALDH1A3* (16 folds) (Fig. 6D). *SOX10* expression is controlled by *MITF* ^41^, which was also found to be downregulated (Fig. 4D).

### AC ablation causes growth arrest and decreases malignancy

**Figure 7:**
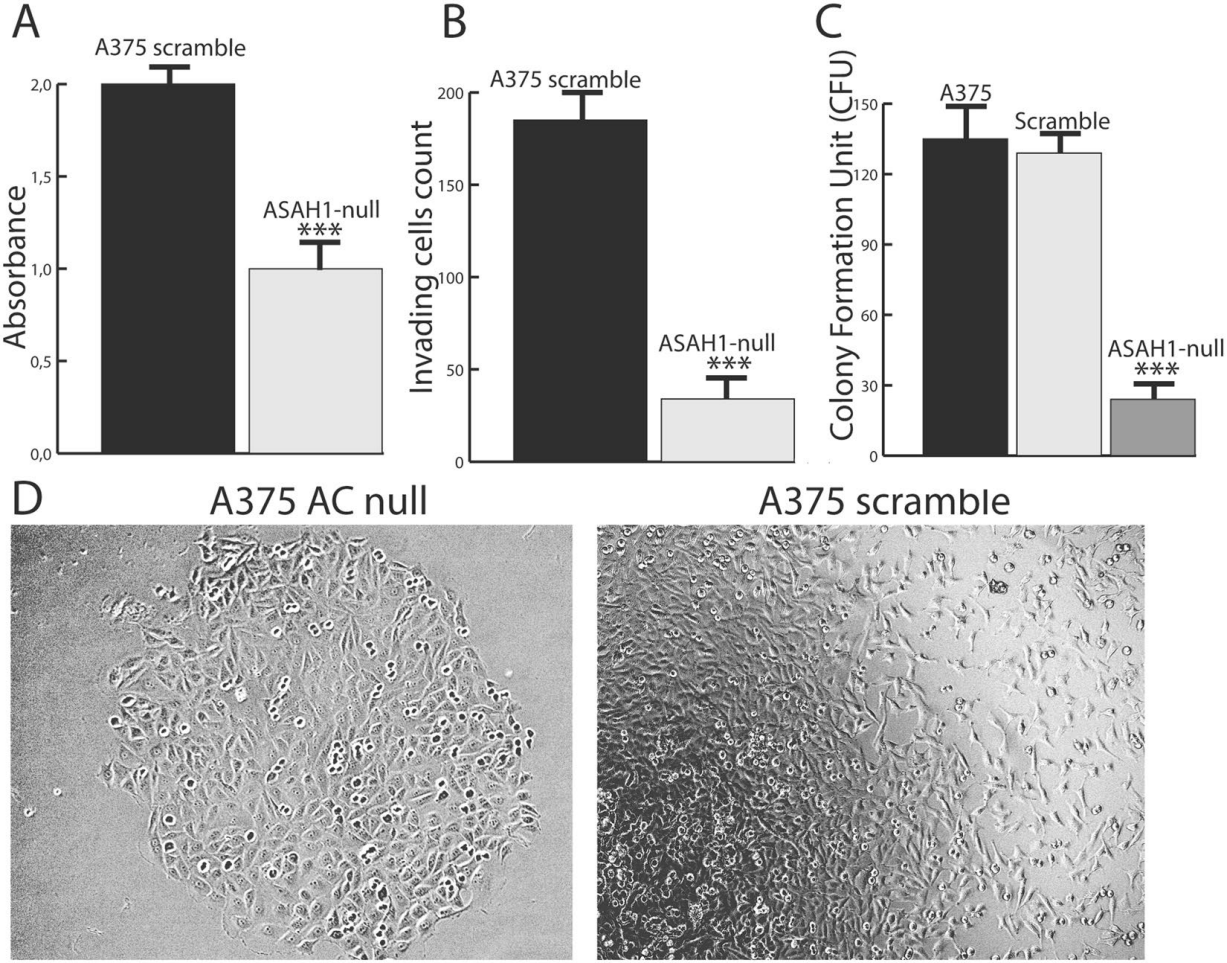
AC deletion causes growth arrest and decreases malignancy. (**A**) Proliferation assay in control (scramble-treated) and *ASAH1*-null A375 cells. Results are expressed as mean ± SEM, n = 12, *p < 0.05, **p < 0,01,***p < 0.001, Student’s *t* test.(**B**) Invasion assay showing *ASAH1*-null cells that have decreased ability to invade using fetal bovine serum as chemoattractant. Results are expressed as mean ± SEM, n = 12, *P < 0.05, **p < 0,01,***P < 0.001; Student’s *t* test. (**C**) Soft Agar assay showing a marked reduction in colony formation of *ASAH1*-null compared to control cells (solid bar: scramble treated; empty bar: naïve). Results are expressed as mean ± SEM, n = 10, with each experiment performed in two independent groups of cells. *p < 0.05, ** p < 0,01,***p < 0.001, Student’s *t* test. (**D**) Images taken 10 days after treatment demonstrating growth reduction in *ASAH1*-null cells (left) compared to control scramble-treated cells (right). Cell invasion and soft-agar assays were assessed 30 days after RT-PCR screening. Proliferation was evaluated 17 days after ASAH1 gene ablation.

Proliferation assays showed that *ASAH1*-null cells have a slower replication rate compared to naïve or scrambled-transfected cells (Fig. 7A,D). Moreover, *ASAH1*-null cells showed a markedly reduced ability to invade (Fig. 7B), and to form colonies in soft agar (p < 0.01, Fig. 7C). The results suggest that *ASAH1* and its encoded AC protein are crucial to maintain cancer cell malignancy.

### AC ablation alters ceramide levels

**Figure 8:**
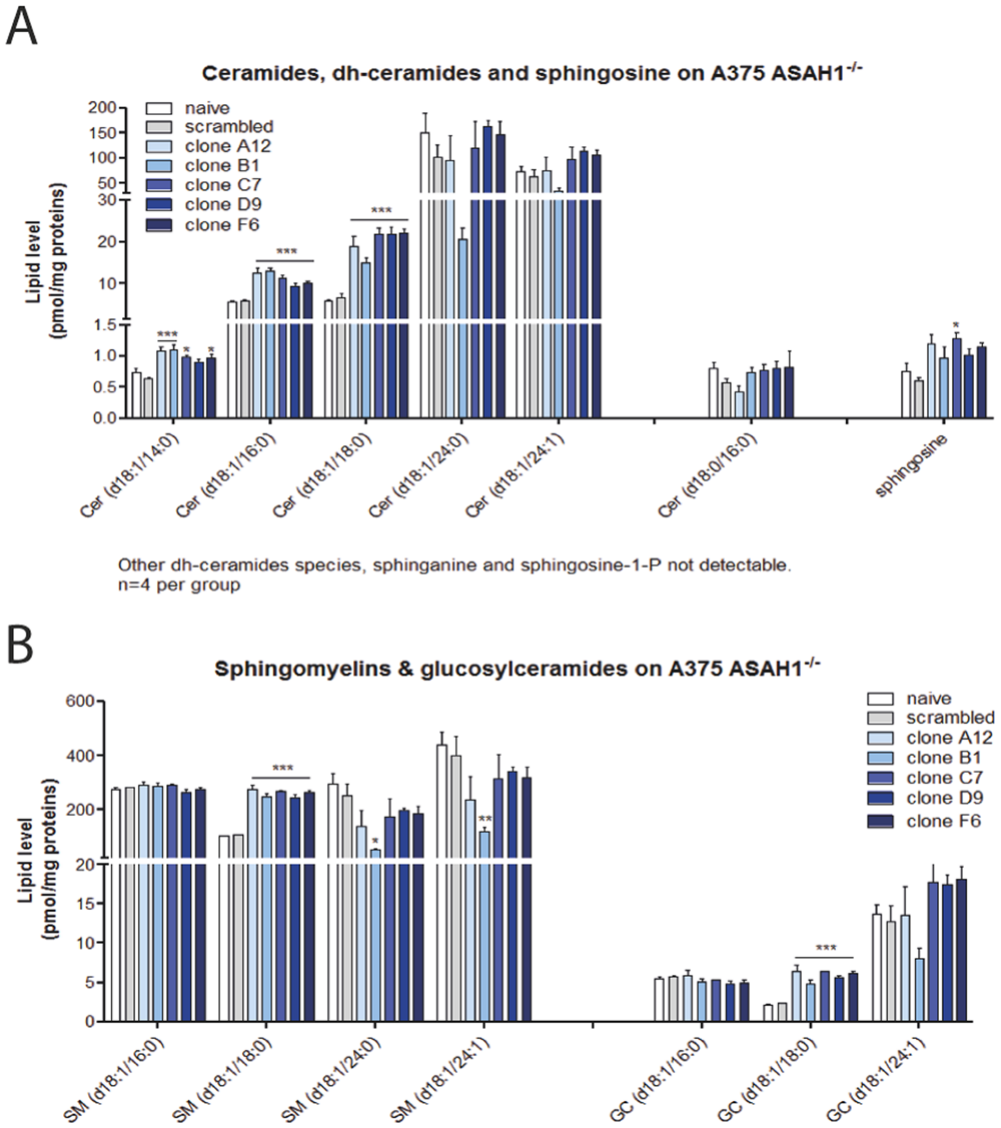
AC deletion alters the sphingolipidome of A375 melanoma cells. (**A**) Levels of medium-chain ceramides (C14:0, C16:0 and C18:0), dihydroceramides and sphingosine in various clones of control and *ASAH1*-null cells. (**B**) Levels of sphingomyelins and glucosylceramides in the same clones. Results are expressed as mean ± SEM (n = 3), with each experiment performed in two independent groups of cells. *p < 0.05, **p < 0.01, one-way ANOVA followed by Tukey’s test. Experiments were performed on pellets obtained 20 days after ASAH1 ablation and repeated three times.

We used a targeted lipidomics approach^32^ to assess the impact of AC deletion on the sphingolipidome of A375 cells. Compared to control cells (naïve or scramble-treated), all *ASAH1*-null clones showed significantly greater accumulation of long-chain saturated ceramides (C14:0, C16:0 and C18:0 ceramides, Fig. 8A), which are preferred substrates for AC activity. By contrast, no significant differences were seen in longer-chain saturated or unsaturated ceramides that are not cleaved by AC (C24:0 and C24:1). The levels of dihydroceramides [cer(d18:0/16:0)] were not affected, whereas slight non-significant increases were noted in the levels of sphingosine, possibly due to compensative effects by other enzymes^19^. An increase in the accumulation of sphingomyelins and hexosylceramides containing 18:0 ceramide chain was also observed (Fig. 8B).

### AC recovery partially restores the A735 phenotype

**Figure 9:**
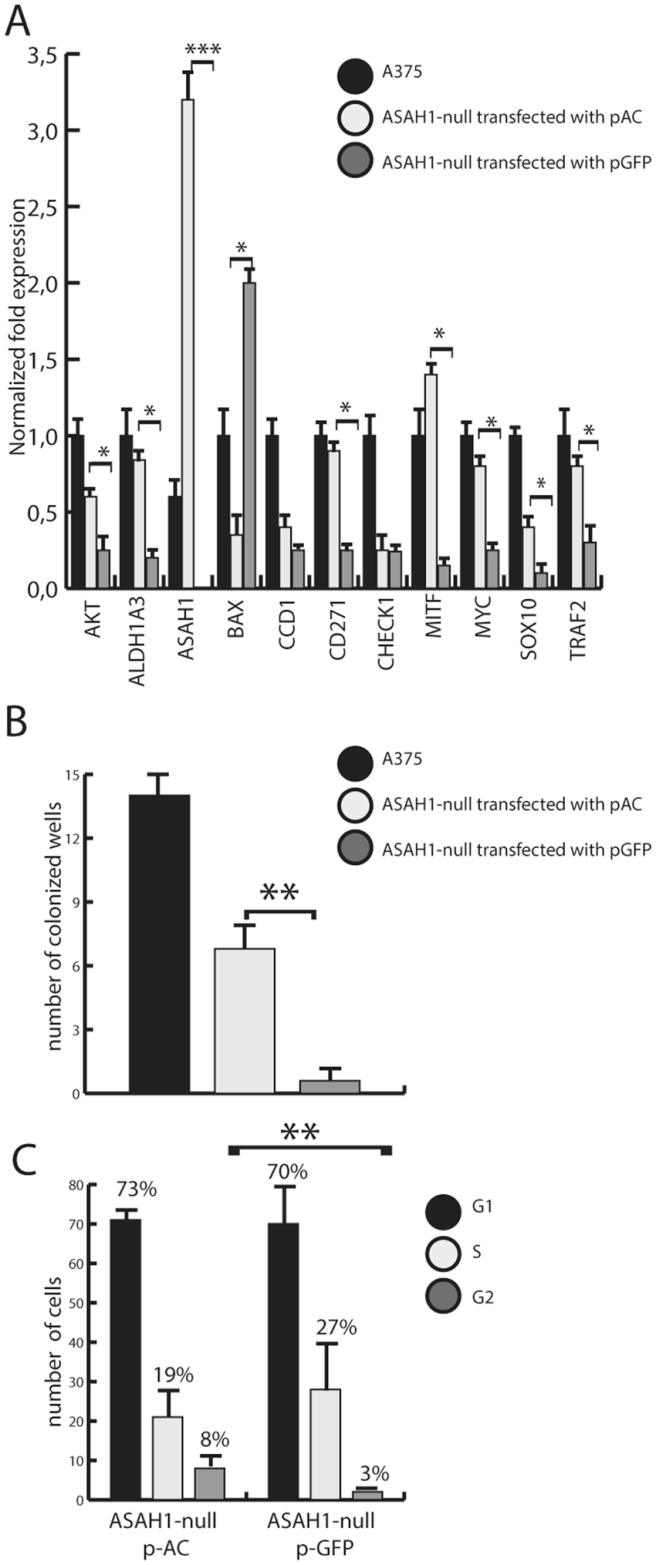
AC transfection partially restores phenotype in AC-null A735 cells. (**A**) Real-Time PCR performed 2 days after transfection with pASAH1 shows, as expected, a strong over-expression of *ASAH1* and a complete recovery of *ALDH1A3*, *BAX*, *CD271*, *MITF* and *MYC* expression. *AKT*, *CCD1*, *SOX10* and *TRAF* showed partial expression recovery. No difference was observed in *CHECK1* expression. Results are expressed as mean ± SEM, n = 3, with each experiment performed in technical and biological triplicates: experiments were performed using three replicates each and repeated three times. *p < 0.05, ** p < 0,01, ***p < 0.001, Student’s *t* test. (**B**) Self-renewal assay in control (scramble-treated), *ASAH1*-null transfected with pASAH1 and ASAH1-null A375 cells show a recovery of self-renewal capabilities of ASAH1-null cells transfected with *ASAH-1* cDNA. Results are expressed as mean ± SEM, n = 3, with each experiment performed in technical and biological triplicates: experiments were performed using three replicates each and repeated three times. *p < 0.05, ** p < 0,01,***p < 0.001, Student’s *t* test. (**C**) The rescue of cell cycle progression was assessed using propidium iodide and flow cytometry analysis. Dot plots show different distribution between G1, S and G2 phases in control (scramble-treated *ASAH1*-null cells) versus *ASAH1*-null cells transfected with pASAH1. *p < 0.05, **p < 0,01,***p < 0.001, Student’s *t* test. Recovery assays were performed on cells that were frozen at −80° in FBS and DMSO, 30 days after the CRISPR/Cas9 ablation.

Lastly, *ASAH1*-null cells were transfected with a plasmid containing the *ASAH1* cDNA. *ASAH1* transfection partially restored expression of many genes altered by CRISPR-mediated *ASAH1* ablation (Fig. 9A). In addition, *ASAH1* transfection increased self-renewal capability of *ASAH1*-null cells (Fig. 9B) and partially rescued them from G1/S blockade (Fig. 9C).

## Discussion

The present study provides the first detailed description of the impact exerted by complete depletion of the *ASAH1* gene, encoding for the lipid amidase AC, in a human melanoma cell line. Previous studies using pharmacological or gene-silencing approaches have linked lowered AC activity to increased apoptosis, senescence and cell growth arrest^2, 25^. However, in these studies complete and permanent AC suppression was never achieved, and the roles of this enzyme in controlling the fate of melanoma cells remain therefore unclear. Here, we used the CrispR/Cas9 system to generate A375 melanoma cells in which AC is totally and stably depleted by a large deletion event in its encoding *ASAH1* gene. The results show that AC ablation perturbs ceramide metabolism and directs A375 cells toward either apoptosis or senescence. Importantly, our findings indicate for the first time that AC deletion deprives A375 cells of the ability to form tumor-initiating cells and causes a dramatic reduction in self-renewal, a result that points to AC blockade as a potential therapeutic option for the treatment of metastatic melanoma, in which cancer initiating cells are thought to play an obligatory role^39, 42^. These findings are also independently supported by a recent study showing that prostate cancer cells, with lowered levels of AC, are less prone to proliferate and metastasize *in vivo* ^43^. To confirm this evidence, the upregulation of AC was related to the escape process from radiotherapy-induced apoptosis of prostate cancer cells^44^.

As expected from prior work^19^, AC ablation causes substantial changes in the cellular sphingolipidome. We found that *ASAH1*-null cells accumulate abnormally high levels of ceramides that are preferred substrates for AC (C14:0, C16:0 and C18:0). By contrast, no differences were seen in longer-chain saturated or unsaturated ceramides that are not recognized by this enzyme (e.g. 24:0 and 24:1). While substantial, ceramide changes following complete AC ablation were comparable to those observed in A375 cells treated with pharmacological inhibitors such as ARN080^19^ or ARN14988^8^ in which blockade of AC activity was only partial. There are two non-exclusive explanations for this finding. The first is that adaptive alterations in sphingolipid metabolism, e.g. in *de novo* synthesis of ceramides, might compensate for the complete removal of AC. A second possibility is that different cell states might be associated with different levels of ceramide accumulation. Consistent with this view, we found that AC ablation forces A375 cells toward three mutually exclusive fates – apoptosis, senescence or growth arrest – which are represented in the same clones but are likely to be associated with distinct ceramide concentrations. The lipid profile of each clone would represent the algebraic summation of the profiles associated with each of those states.

**Figure 10:**
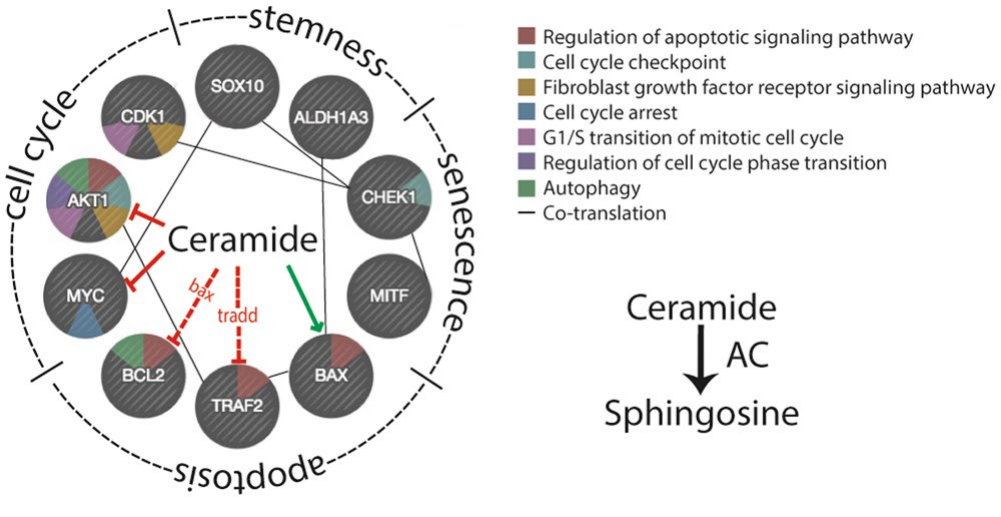
Regulation of cell fate by ceramide. Hypothetic model showing potential interactions of ceramide with various factors involved in cell cycle, stemness, senescence and apoptosis.

Our results show that AC ablation strongly perturbs the rate of proliferation, growth and invasiveness of A375 cells. These alterations are accompanied by marked transcriptional changes in genes involved in those processes. As illustrated in Fig. 10, ceramide is connected to those genes through a network of interactions that can influence cellular fate in profound ways. Previous pharmacological and gene-silencing experiments have suggested that AC participates in the control of cancer cell proliferation and malignancy^45, 46^. In the present report, the complete suppression of AC expression was found to cause a stronger reduction in proliferation and invasion capabilities compared to previous studies^20^. As described, the cell-permeant ceramide analog C6 causes cell cycle arrest in the G1/S phase^25^. We found the same phenomenon in *ASAH1*-null cells, of which only 4% enter the G2 phase, compared to 25% in control cells. The mechanism underlying this effect is unknown, but is likely to involve the downregulation of *MYC*, *CDK1*, *CHK1* and *AKT*. These genes are part of a regulatory network that is critical for G1/S transition and for coordinating S-G2-M progression^36^. *MYC* and *AKT* are also directly regulated by ceramide (Fig. 10), which suppresses their activity^47, 48^. Because *AKT* also regulates autophagy and AC over-expression increases this process, in concert with lysosomal density^49^ it is tempting to speculate that *ASAH1*-null cells may display a decreased degree of autophagy and might be therefore less prone to the “insult-ready” phenotype described by Liu *et al*.^49^.

Ceramide plays a crucial role in the regulation of apoptosis^49^. Consistent to this view, we found that 22% of *ASAH1*-null cells undergo apoptosis within the first week in culture. As this experiment was conducted on clonal populations, it suggests that cells derived from the same clone react differently to the absence of AC. It is possible, indeed, that AC ablation may lead to stochastic perturbations in lipid profile, which might lead to different cell fates. Testing this hypothesis will require additional experimentations. RT-PCR studies elucidated the activation of various pathways involved in the regulation of apoptosis. The pro-apoptotic factor *BAX*, which is induced by ceramide^38^, is upregulated in *ASAH1*-null cells. As shown in Fig. 10, *BAX* interacts with *BCL2*, interfering with the pro-survival pathway regulated by this factor^50^. This interaction leads to a cascade of events in which cytochrome c is released from mitochondria due to a loss in membrane potential^51^. On the other hand, the anti-apoptotic factor *TRAF2* is downregulated following AC ablation. *TRAF2* interacts with TNF receptors and functions as a mediator of the anti-apoptotic signals emanated from these receptors^52^. *TRAF2* downregulation might activate NF-kB and pro-inflammatory events of TNF-α^53^.

Ceramide levels increase when cells enter senescence^22^. We observed that the fraction of *ASAH1*-null cells that did not become apoptotic (38%) were positive to the senescence-associated marker ß-galactosidase^54^. This was accompanied by a profound downregulation of *MITF*, a transcription factor that controls melanocyte differentiation and development^55^. Decreased *MITF* expression has been described in senescent melanocytes and its downregulation has been shown to promote senescence in melanoma cells^56^.

The most surprising and, possibly, most important finding of the present study is that cancer-initiating cell generation is virtually abolished in *ASAH1*-null cells. This striking effect is associated with a marked suppression of two stemness markers, *CD271* and *SOX10*, the latter of which is also known to enhance *MITF* transcription^57^. These results suggest that AC blockade interferes with self-renewal and cancer-initiating cell generation in the highly invasive A375 melanoma cell line. Downregulation of *ALDH1A3*, which encodes for the most abundant aldehyde dehydrogenase isoform present in melanoma, suggests a weakened response to detoxification^58^. *ALDH1A3* is involved in cellular detoxification, differentiation and drug resistance through the oxidation of cellular aldehydes^59^. Moreover, *ALDH1A3* is a marker of normal and malignant melanoma and a predictor of poor clinical outcome in this form of cancer^58^. The observed ablation of cancer-initiating cell formation and self-renewal appears, in conclusion, to be dependent on AC and suggests that AC inhibitors may find therapeutic use as adjuvant therapy in advanced melanoma.

## Electronic supplementary material

Supplementary Table 1

## References

[1] Mao C, Obeid LM (2008). Ceramidases: regulators of cellular responses mediated by ceramide, sphingosine, and sphingosine-1-phosphate. Biochim Biophys Acta.

[2] Alphonse G (2002). Ceramide induces activation of the mitochondrial/caspases pathway in Jurkat and SCC61 cells sensitive to gamma-radiation but activation of this sequence is defective in radioresistant SQ20B cells. Int J Radiat Biol.

[3] Abuhusain HJ (2013). A metabolic shift favoring sphingosine 1-phosphate at the expense of ceramide controls glioblastoma angiogenesis. J Biol Chem.

[4] Beckham TH (2013). Acid ceramidase induces sphingosine kinase 1/S1P receptor 2-mediated activation of oncogenic Akt signaling. Oncogenesis.

[5] Bedia C, Casas J, Andrieu-Abadie N, Fabrias G, Levade T (2011). Acid ceramidase expression modulates the sensitivity of A375 melanoma cells to dacarbazine. J Biol Chem.

[6] Beckham TH (2013). Acid ceramidase promotes nuclear export of PTEN through sphingosine 1-phosphate mediated Akt signaling. PLoS One.

[7] Saad AF (2007). The functional effects of acid ceramidase overexpression in prostate cancer progression and resistance to chemotherapy. Cancer Biol Ther.

[8] Realini, N. *et al*. Acid Ceramidase in Melanoma: Expression, Localization and Effects of Pharmacological Inhibition. *J Biol Chem* (2015). 10.1074/jbc.M115.666909 PMC4732224 26553872

[9] Seelan RS (2000). Human acid ceramidase is overexpressed but not mutated in prostate cancer. Genes Chromosomes Cancer.

[10] Park JH, Schuchman EH (2006). Acid ceramidase and human disease. Biochim Biophys Acta.

[11] Zeidan YH (2008). Molecular targeting of acid ceramidase: implications to cancer therapy. Curr Drug Targets.

[12] Hajj C, Becker-Flegler KA, Haimovitz-Friedman A (2015). Novel mechanisms of action of classical chemotherapeutic agents on sphingolipid pathways. Biol Chem.

[13] Galadari S, Rahman A, Pallichankandy S, Thayyullathil F (2015). Tumor suppressive functions of ceramide: evidence and mechanisms. Apoptosis.

[14] Dany M, Ogretmen B (2015). Ceramide induced mitophagy and tumor suppression. Biochim Biophys Acta.

[15] Mahdy AE (2009). Acid ceramidase upregulation in prostate cancer cells confers resistance to radiation: AC inhibition, a potential radiosensitizer. Mol Ther.

[16] Gouaze-Andersson V (2011). Inhibition of acid ceramidase by a 2-substituted aminoethanol amide synergistically sensitizes prostate cancer cells to N-(4-hydroxyphenyl) retinamide. Prostate.

[17] Elojeimy S (2007). Role of acid ceramidase in resistance to FasL: therapeutic approaches based on acid ceramidase inhibitors and FasL gene therapy. Mol Ther.

[18] Morales A (2007). Pharmacological inhibition or small interfering RNA targeting acid ceramidase sensitizes hepatoma cells to chemotherapy and reduces tumor growth *in vivo*. Oncogene.

[19] Realini N (2013). Discovery of highly potent acid ceramidase inhibitors with *in vitro* tumor chemosensitizing activity. Sci Rep.

[20] Berndt N, Patel R, Yang H, Balasis ME, Sebti SM (2013). Akt2 and acid ceramidase cooperate to induce cell invasion and resistance to apoptosis. Cell Cycle.

[21] Feng H (2014). EGFR phosphorylation of DCBLD2 recruits TRAF6 and stimulates AKT-promoted tumorigenesis. J Clin Invest.

[22] Venable ME, Lee JY, Smyth MJ, Bielawska A, Obeid LM (1995). Role of ceramide in cellular senescence. J Biol Chem.

[23] Bieberich E (2008). Ceramide signaling in cancer and stem cells. Future Lipidol.

[24] Eliyahu E, Park JH, Shtraizent N, He X, Schuchman EH (2007). Acid ceramidase is a novel factor required for early embryo survival. FASEB J.

[25] Hannun YA, Obeid LM (2008). Principles of bioactive lipid signalling: lessons from sphingolipids. Nat Rev Mol Cell Biol.

[26] Santini R (2014). SOX2 regulates self-renewal and tumorigenicity of human melanoma-initiating cells. Oncogene.

[27] Chen X (2014). Dual sgRNA-directed gene knockout using CRISPR/Cas9 technology in Caenorhabditis elegans. Sci Rep.

[28] Lai M (2016). Gene editing of DNAH11 restores normal cilia motility in primary ciliary dyskinesia. J Med Genet.

[29] Ran FA (2013). Genome engineering using the CRISPR-Cas9 system. Nat Protoc.

[30] Chu VT (2015). Increasing the efficiency of homology-directed repair for CRISPR-Cas9-induced precise gene editing in mammalian cells. Nat Biotechnol.

[31] Bligh EG, Dyer WJ (1959). A rapid method of total lipid extraction and purification. Can J Biochem Physiol.

[32] Basit A, Piomelli D, Armirotti A (2015). Rapid evaluation of 25 key sphingolipids and phosphosphingolipids in human plasma by LC-MS/MS. Anal Bioanal Chem.

[33] Obeid LM, Linardic CM, Karolak LA, Hannun YA (1993). Programmed cell death induced by ceramide. Science.

[34] Venable ME, Yin X (2009). Ceramide induces endothelial cell senescence. Cell Biochem Funct.

[35] Wellbrock C (2008). Oncogenic BRAF regulates melanoma proliferation through the lineage specific factor MITF. PLoS One.

[36] Bartek J, Lukas J (2001). Mammalian G1- and S-phase checkpoints in response to DNA damage. Curr Opin Cell Biol.

[37] Astarita G (2015). Methamphetamine accelerates cellular senescence through stimulation of de novo ceramide biosynthesis. PLoS One.

[38] Kim R, Minami K, Nishimoto N, Toge T (2001). Enhancement of antitumor effect by intratumoral administration of bax gene in combination with anticancer drugs in gastric cancer. Int J Oncol.

[39] Lee N, Barthel SR, Schatton T (2014). Melanoma stem cells and metastasis: mimicking hematopoietic cell trafficking?. Lab Invest.

[40] Ozsvari B, Lamb R, Lisanti MP (2016). Repurposing of FDA-approved drugs against cancer - focus on metastasis. Aging (Albany NY).

[41] Potterf SB, Furumura M, Dunn KJ, Arnheiter H, Pavan WJ (2000). Transcription factor hierarchy in Waardenburg syndrome: regulation of MITF expression by SOX10 and PAX3. Hum Genet.

[42] Boiko AD (2010). Human melanoma-initiating cells express neural crest nerve growth factor receptor CD271. Nature.

[43] Camacho L (2013). Acid ceramidase as a therapeutic target in metastatic prostate cancer. J Lipid Res.

[44] Cheng JC (2013). Radiation-induced acid ceramidase confers prostate cancer resistance and tumor relapse. The Journal of clinical investigation.

[45] Li CM (2002). Insertional mutagenesis of the mouse acid ceramidase gene leads to early embryonic lethality in homozygotes and progressive lipid storage disease in heterozygotes. Genomics.

[46] Roh JL, Park JY, Kim EH, Jang HJ (2016). Targeting acid ceramidase sensitises head and neck cancer to cisplatin. Eur J Cancer.

[47] Stratford S, Hoehn KL, Liu F, Summers SA (2004). Regulation of insulin action by ceramide: dual mechanisms linking ceramide accumulation to the inhibition of Akt/protein kinase B. J Biol Chem.

[48] Sultan I (2006). Regulation of the sphingosine-recycling pathway for ceramide generation by oxidative stress, and its role in controlling c-Myc/Max function. Biochem J.

[49] Turner LS (2011). Autophagy is increased in prostate cancer cells overexpressing acid ceramidase and enhances resistance to C6 ceramide. Prostate Cancer Prostatic Dis.

[50] Teni T, Pawar S, Sanghvi V, Saranath D (2002). Expression of bcl-2 and bax in chewing tobacco-induced oral cancers and oral lesions from India. Pathol Oncol Res.

[51] Heise, T. *et al*. The La protein counteracts cisplatin-induced cell death by stimulating protein synthesis of anti-apoptotic factor Bcl2. *Oncotarget* (2016). 10.18632/oncotarget.8819 PMC5045424 27105491

[52] Borghi, A., Verstrepen, L. & Beyaert, R. TRAF2 multitasking in TNF receptor-induced signaling to NF-kappaB, MAP kinases and cell death. *Biochem Pharmacol* (2016). 10.1016/j.bcp.2016.03.009 26993379

[53] Zhang L (2016). TRAF2 exerts opposing effects on basal and TNFalpha-induced activation of the classic IKK complex in hematopoietic cells in mice. J Cell Sci.

[54] Lee BY (2006). Senescence-associated beta-galactosidase is lysosomal beta-galactosidase. Aging Cell.

[55] Levy C, Khaled M, Fisher DE (2006). MITF: master regulator of melanocyte development and melanoma oncogene. Trends Mol Med.

[56] Giuliano S (2010). Microphthalmia-associated transcription factor controls the DNA damage response and a lineage-specific senescence program in melanomas. Cancer Res.

[57] Bondurand N (2000). Interaction among SOX10, PAX3 and MITF, three genes altered in Waardenburg syndrome. Hum Mol Genet.

[58] Luo Y (2012). ALDH1A isozymes are markers of human melanoma stem cells and potential therapeutic targets. Stem Cells.

[59] Shao C (2014). Essential role of aldehyde dehydrogenase 1A3 for the maintenance of non-small cell lung cancer stem cells is associated with the STAT3 pathway. Clin Cancer Res.

