# Supplementary material for: Complete Acid Ceramidase ablation prevents cancer-initiating cell formation in melanoma cells
Source: Sci Rep. 2017 Aug 7;7:7411. doi: 10.1038/s41598-017-07606-w (PMC5547127; doi:10.1038/s41598-017-07606-w)
Supplement: Supplementary file 1 — Supplementary Table 1 [file 41598_2017_7606_MOESM1_ESM.pdf]

Complete Acid Ceramidase ablation prevents cancer-initiating cell formation in melanoma cells – Supplementary Table 1

Michele Lai[1234], Natalia Realini[1], Marco La Ferla[2], Ilaria Passalacqua[3], Giulia Matteoli[3]

Anand Ganesan[5], Mauro Pistello[3], Chiara Maria Mazzanti[2*], Daniele Piomelli[4*]

1 Drug Discovery and Development, Istituto Italiano di Tecnologia, Genova 16163, Italy

2 Pisa Science Foundation, Pisa, Italy 56121

3 Retrovirus Center and Virology Section, Department of Translational Research, University of Pisa, Pisa, Italy

4 Departments of Anatomy and Neurobiology, Pharmacology and Biological Chemistry, University of California, Irvine, California 92617

5 Department of Dermatology, University of California, Irvine, California 92868

Gene Tables

Apoptosis Panel

Array Layout

ABL1<br>A01	AIFM1<br>A02	AKT1<br>A03	APAF1<br>A04	BAD<br>A05	BAG1<br>A06	BAG3<br>A07	BAK1<br>A08	BAX<br>A09	BCL10<br>A10	BCL2<br>A11	BCL2A1<br>A12
BCL2L1<br>B01	BCL2L10<br>B02	BCL2L11<br>B03	BCL2L2<br>B04	BFAR<br>B05	BID<br>B06	BIK<br>B07	BIRC2<br>B08	BIRC3<br>B09	BIRC5<br>B10	BIRC6<br>B11	BNIP2<br>B12
BNIP3<br>C01	BNIP3L<br>C02	BRAF<br>C03	CASP1<br>C04	CASP10<br>C05	CASP14<br>C06	CASP2<br>C07	CASP3<br>C08	CASP4<br>C09	CASP5<br>C10	CASP6<br>C11	CASP7<br>C12
CASP8<br>D01	CASP9<br>D02	CD27<br>D03	CD40<br>D04	CD40LG<br>D05	CD70<br>D06	CFLAR<br>D07	CIDEA<br>D08	CIDEB<br>D09	CRADD<br>D10	CYCS<br>D11	DAPK1<br>D12
DFFA<br>E01	DIABLO<br>E02	FADD<br>E03	FAS<br>E04	FASLG<br>E05	GADD45A<br>E06	HRK<br>E07	IGF1R<br>E08	IL10<br>E09	LTA<br>E10	LTBR<br>E11	MCL1<br>E12
NAIP<br>F01	NFKB1<br>F02	NOD1<br>F03	NOL3<br>F04	PYCARD<br>F05	RIPK2<br>F06	TNF<br>F07	TNFRSF10A<br>F08	TNFRSF10B<br>F09	TNFRSF11B<br>F10	TNFRSF1A<br>F11	TNFRSF1B<br>F12
TNFRSF21<br>G01	TNFRSF25<br>G02	TNFRSF9<br>G03	TNFSF10<br>G04	TNFSF8<br>G05	TP53<br>G06	TP53BP2<br>G07	TP73<br>G08	TRADD<br>G09	TRAF2<br>G10	TRAF3<br>G11	XIAP<br>G12
ACTB<br>H01	B2M<br>H02	GAPDH<br>H03	HPRT1<br>H04	RPLP0<br>H05	HGDC<br>H06	RTC<br>H07	RTC<br>H08	RTC<br>H09	PPC<br>H10	PPC<br>H11	PPC<br>H12

Senescence Panel

ABL1<br>A01	AKT1<br>A02	ALDH1A3<br>A03	ATM<br>A04	BMI1<br>A05	CALR<br>A06	CCNA2<br>A07	CCNB1<br>A08	CCND1<br>A09	CCNE1<br>A10	CD44<br>A11	CDC25C<br>A12
CDK2<br>B01	CDK4<br>B02	CDK6<br>B03	CDKN1A<br>B04	CDKN1B<br>B05	CDKN1C<br>B06	CDKN2A<br>B07	CDKN2B<br>B08	CDKN2C<br>B09	CDKN2D<br>B10	CHEK1<br>B11	CHEK2<br>B12
CITED2<br>C01	COL1A1<br>C02	COL3A1<br>C03	CREG1<br>C04	E2F1<br>C05	E2F3<br>C06	EGR1<br>C07	ETS1<br>C08	ETS2<br>C09	FN1<br>C10	GADD45A<br>C11	GLB1<br>C12
GSK3B<br>D01	HRAS<br>D02	ID1<br>D03	IFNG<br>D04	IGF1<br>D05	IGF1R<br>D06	IGFBP3<br>D07	IGFBP5<br>D08	IGFBP7<br>D09	ING1<br>D10	IRF3<br>D11	IRF5<br>D12
IRF7<br>E01	MAP2K1<br>E02	MAP2K3<br>E03	MAP2K6<br>E04	MAPK14<br>E05	MDM2<br>E06	MORC3<br>E07	MYC<br>E08	NBN<br>E09	NFKB1<br>E10	NOX4<br>E11	PCNA<br>E12
PIK3CA<br>F01	PLAU<br>F02	PRKCD<br>F03	PTEN<br>F04	RB1<br>F05	RBL1<br>F06	RBL2<br>F07	SERPINB2<br>F08	SERPINE1<br>F09	SIRT1<br>F10	SOD1<br>F11	SOD2<br>F12
SPARC<br>G01	TBX2<br>G02	TBX3<br>G03	TERF2<br>G04	TERT<br>G05	TGFB1<br>G06	TGFB1I1<br>G07	THBS1<br>G08	TP53<br>G09	TP53BP1<br>G10	TWIST1<br>G11	VIM<br>G12
B2M<br>H01	HPRT1<br>H02	RPL13A<br>H03	GAPDH<br>H04	ACTB<br>H05	HGDC<br>H06	RTC<br>H07	RTC<br>H08	RTC<br>H09	PPC<br>H10	PPC<br>H11	PPC<br>H12
